# Control of wrist movement in deafferented man: evidence for a mixed strategy of position and amplitude control

**DOI:** 10.1007/s00221-017-5066-5

**Published:** 2017-08-18

**Authors:** R. Chris Miall, P. Haggard, J. D. Cole

**Affiliations:** 1 School of Psychology, University of Birmingham, Birmingham, B15 2TT UK; 2 Institute of Cognitive Neuroscience, University College London, Queen Square, London, WC1N 3AR UK; 3 Centre of Postgraduate Research and Education, Bournemouth University, Bournemouth, UK

**Keywords:** Motor control, Wrist movement, Proprioception, Amplitude, End-point, Equilibrium position

## Abstract

There is a continuing debate about control of voluntary movement, with conflicted evidence about the balance between control of movement vectors (amplitude control) that implies knowledge of the starting position for accuracy, and equilibrium point or final position control, that is independent of the starting conditions. We tested wrist flexion and extension movements in a man with a chronic peripheral neuronopathy that deprived him of proprioceptive knowledge of his wrist angles. In a series of experiments, we demonstrate that he could scale the amplitude of his wrist movements in flexion/extension, even without visual feedback, and appeared to adopt a strategy of moving via a central wrist position when asked to reach target angles from unknown start locations. When examining the relationship between positional error at the start and end of each movement in long sequences of movements, we report that he appears to have three canonical positions that he can reach relatively successfully, in flexion, in extension and in the centre. These are consistent with end-point or position control. Other positions were reached with errors that suggest amplitude control. Recording wrist flexor and extensor EMG confirmed that the flexion and extension canonical positions were reached by strong flexor and extensor activity, without antagonist activity, and other positions were reached with graded muscle activation levels. The central canonical position does not appear to be reached by either maximal co-contraction or by complete relaxation, but may have been reached by matched low-level co-contraction.

## Introduction

There are two major alternative theories of voluntary motor control that, despite many papers addressing their differences, are still debated. The first is that the central nervous system controls the vector of a movement, with motor signals that command changes in muscle force to move the motor system from its current state through to a new desired state (Bock and Eckmiller 1986; Ghez et al. 1995). The alternative view is that the motor system controls the muscle activation thresholds and length–tension characteristics appropriate for the desired final position of the motor system, and then relies on the intrinsic properties of the musculo-skeletal system, enhanced by spinal reflex processes, to shift the motor effector into the desired state (Bizzi et al. 1976; Feldman 1986).

In the limit, and acknowledging simplifications (Feldman and Latash 2004), one can consider the first of these two control strategies as being fully dependent on knowledge of the current position of the motor system to develop commands to shift from the current position to the final position, while the other strategy is independent of information about the starting position, needing only to know the appropriate muscle activation parameters to hold the effector in the desired final position.

From this simplistic viewpoint, and considering point-to-point movement around a single joint, one can then describe the two processes as ‘amplitude’ control versus final (target position) or ‘end-point’ control. These may be extremes on a graded continuum of control [see Graaff et al. (2016) for a summary of the literature], but in this paper we will use the terms amplitude and position control as a verbal shorthand for control biased towards the former and the latter, respectively.

To gain more understanding of these processes, several groups have studied experimental animals or neuropathological cases where the proprioceptive system has been lost. This then allows test of the control of movement in conditions where the current state of their motor system is unknown. If movements are performed adequately in this circumstance, despite uncertainty of the starting position, it provides evidence for motor strategies based on final position control; if movements cannot be achieved under these circumstances, and specifically if the intended movement amplitude is produced without taking account of starting position, then this provides evidence for amplitude control. In an early study of monkeys surgically deprived of limb sensation, Polit and Bizzi (1979) provided evidence that they argued supported position control. In particular the monkeys reached the final target position despite mechanical perturbations during the movement that pushed the arm towards or away from the final location. Nougier et al. (1996) also explored this question, testing two deafferented human participants and concluded the opposite, that there was instead evidence for amplitude control.

Most recently, Graaff et al. (2016) and Marini et al. (2016) returned to this question, in neurologically normal participants to explore the balance between these two extremes of control. Graaf et al. argued for beneficial performance of arm reaching movements that repeated an end position, i.e., positional coding of wrist joint angle, whereas Marini et al. argued that their evidence supported vectorial or amplitude coding.

In the light of these recent papers, we present a series of experiments aimed at exploring these questions further. Comparing a subject with chronic peripheral sensory neuronopathy, IW, and neurologically intact participants, we have tested wrist flexion and extension movements in the absence of visual feedback, in conditions in which IW had no proprioceptive or visual feedback of wrist movement. Testing individuals without functional somatosensory feedback offers a rare opportunity to investigate ‘pure’ efferent coding mechanisms, uninfluenced by closed-loop control. We will discuss what his performance, refined over two decades of deafferentation, can tell us about what the intact motor system, minus proprioceptive feedback, may achieve.

## Experiment 1

### Aim

To test a deafferented subject’s ability to control the amplitude of alternating wrist flexion and extension movements without visual feedback.

### Methods

#### Subject

IW, a man with long-standing peripheral large nerve fibre sensory neuronopathy was studied when aged between 42 and 45 years, about 25 years after his neuronopathy at age 19 [a detailed description of this man is available in Cole and Sedgwick (1992) and Cole (1995)]. He has been shown to have no large myelinated sensory afferents from below the neck at the C3 level. He has no stretch reflexes, no sensation of wrist position or movement, and only residual awareness of upper arm movement that may be based on stretch of neck muscles and skin, that are innovated by surviving nerves above the level of the neuronopathy. If sufficient care is taken to restrict movement to the forearm and wrist, and to ensure the wrist and hand do not strike any surrounding objects, he appears to have no awareness of hand position or movement. In contrast thin fibres were unaffected clinically, so pain and temperature perception are normal and motor nerves were also untouched. EMG is normal. He taught himself to control movement over several years using mental or cognitive control and visual supervision (Cole and Katifi 1991; Cole 2016).

#### Ethics

This and all the following experiments were performed with local ethical approval from Oxford University, and with the subjects’ written informed consent. Some of these results have previously been presented as conference abstracts (Miall et al. 1995b; Cole et al. 1997).

#### Task

**Fig. 1:**
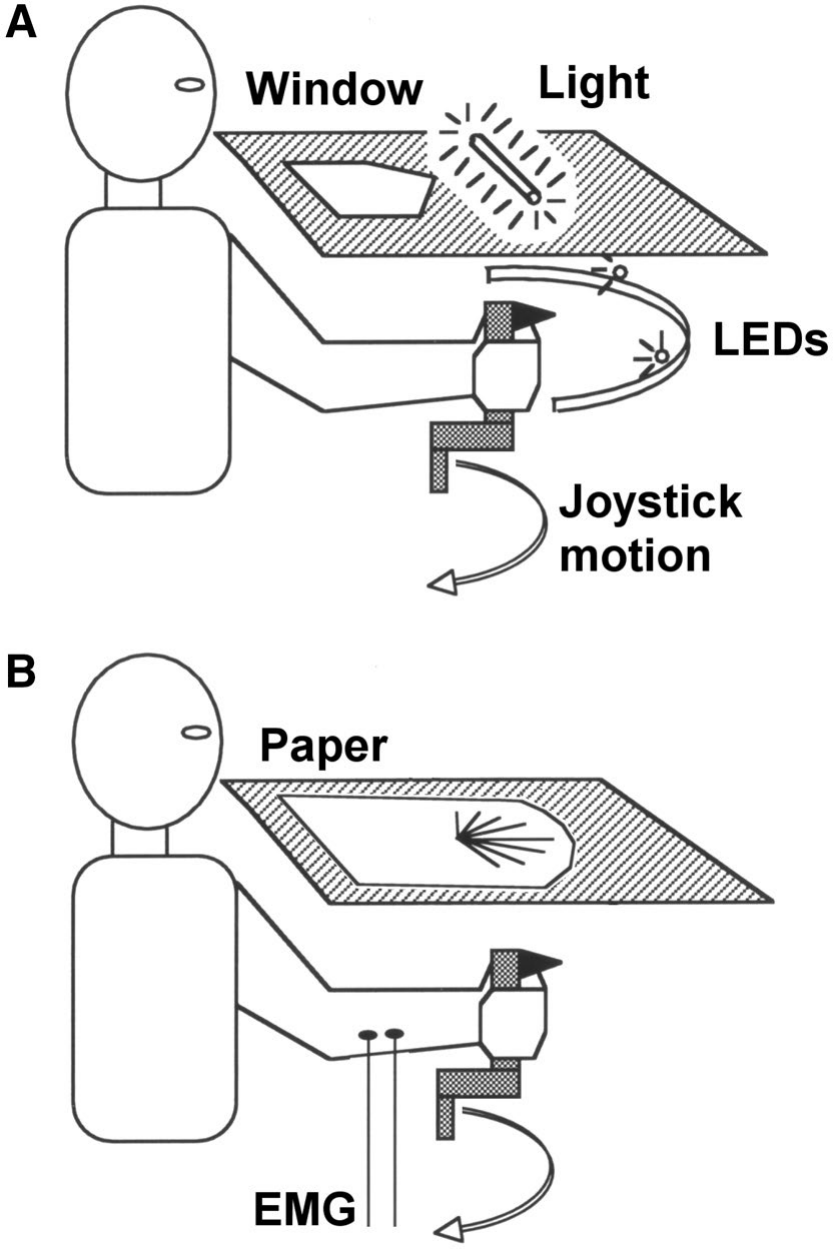
Experimental set-up. **a** The subject could view target LEDs through a window in a horizontal board, but lights above the board and drapes surrounding the board hid the hand and manipulandum from view. **b** For Experiments 4 and 5, a printed paper sheet covered the window, and the subject could see an array of target angles, but had no view of the hand or arm

Subject IW held a light, low friction, plastic manipulandum in his preferred left hand, which allowed unrestricted wrist flexion and extension movements in the horizontal plane. His forearm was firmly supported within a plastic gutter and was restrained by Velcro straps. The forearm was hidden from the subject’s view by a horizontal board (Fig. 1a), with a transparent perspex window inset in the board that was normally covered by a sheet of paper. Light-proof drapes hanging from the plastic board and fastened behind the subject’s neck covered his elbow and upper arm; the drapes were arranged to ensure that they were not visibly moved even during vigorous wrist motion. If the paper sheet covering the window was removed, the subject could view his wrist and hand through the window (although only very dimly illuminated), and also view a circular metal arc centred on the axis of the manipulandum. The arc held two small red LED targets that could be moved by the experimenter to different angular positions, defining the desired amplitude of the wrist movement between the two LED targets. Two small fluorescent light tubes were also mounted on top of the front and rear edge of the perspex window, illuminating the upper surface of the surrounding board. Great care was then taken to ensure that these lighting conditions allowed the subject to see the illuminated LEDs but have no vision of his hand or the manipulandum. The angular position of the manipulandum was monitored by a potentiometer and its voltage signal sampled by a lab computer at 70 Hz with 12-bit precision.

Initially IW was asked to make comfortable wrist flexion and extension movements, without any visual targets. Then the two LEDs were illuminated, separated first by 30° (±15° about the midpoint), then 45° and 60°. IW was asked to flex and extend his wrist to and fro between these visual targets. As he had no visual feedback of his wrist, this was a test of his ability to use visual information about desired amplitude and position to control wrist movement amplitude. IW was tested in this task on one session.

While initially preparing the subject for the task, IW unavoidably gained some visual information about the range of wrist motion available within the device. It was also necessary to give him the opportunity to flex and extend his wrist a couple of times before starting the experiment, so that he was happy with the experimental arrangement. However, he was not able to see the LEDs at this stage, and did not know the wrist motions that we subsequently required of him. The possible rotation of the manipulandum and the extent of the circular frame were nearly 180° whereas the required movements were no more than 60°. In this and all subsequent experiments, the end positions in flexion and extension were not at his anatomic limits of wrist flexion or extension (see “Experiment 5”).

### Results

**Fig. 2:**
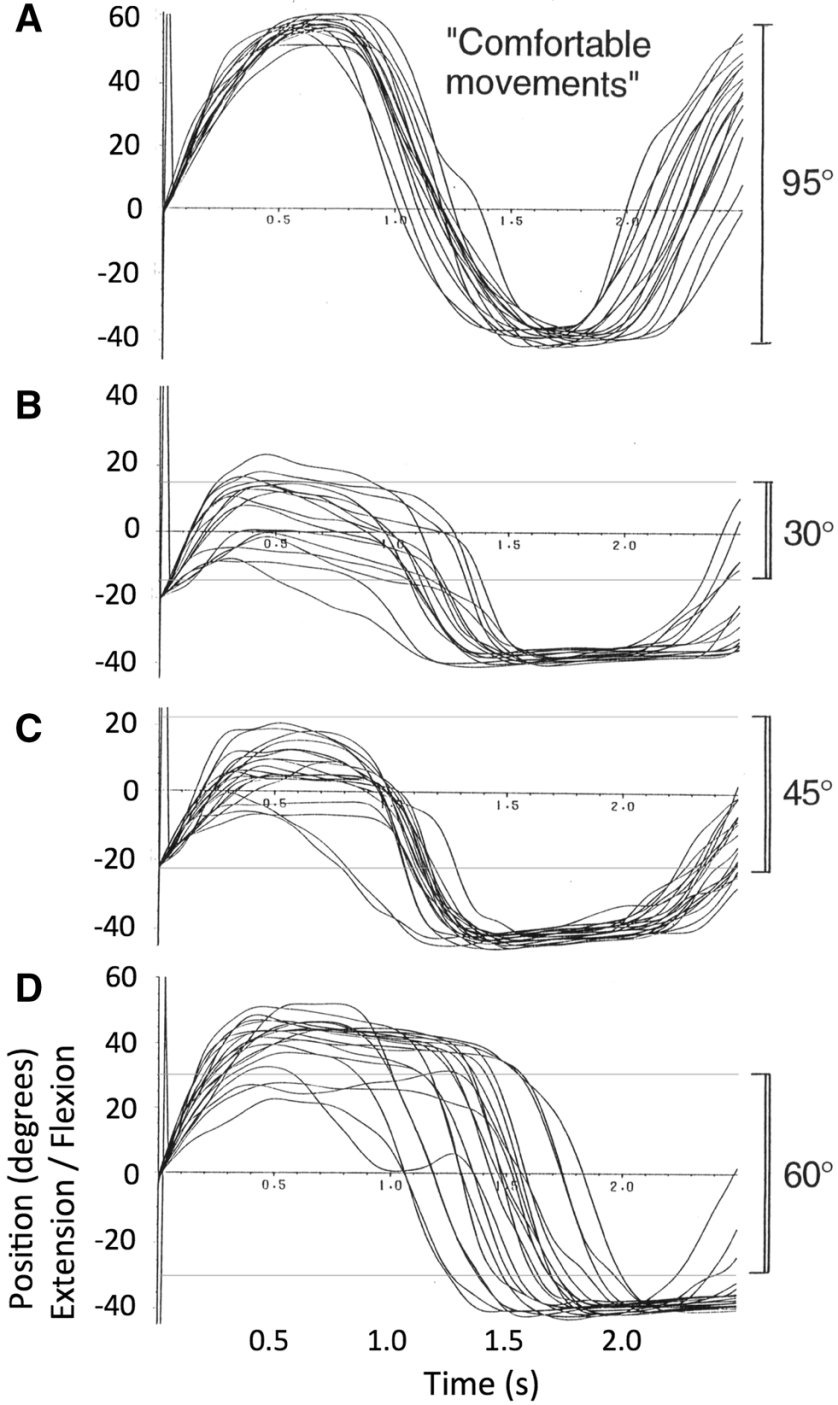
Experiment 1: typical wrist flexion and extension movement made by IW without visual feedback; the *horizontal lines* in **b**–**d** indicate the target positions, set at 30°, 45° and 60°, respectively. Each trace is aligned to the moment the wrist passed through the mean position for the whole run, into flexion. When asked to move to his extreme limits, he reached 80° in flexion and 71° in extension

Typical records are shown in Fig. 2. He was clearly able to scale his free wrist movements on the basis of the visual targets, but tended to overshoot the smaller movements in flexion. His movements were also not symmetrical about the midline, but tended to increase in flexion as the desired movement amplitude increased, while maintaining a nearly constant extension limit with limited variability across trials. The upper panel (Fig. 2a) indicates the position reached when he was initially instructed to make comfortable flexion and extension movements before any visual targets were illuminated. The range of these comfortable movements is about 95°; the extreme range his wrist flexion and extension measured in these experimental conditions, with the same apparatus, was approximately 150° (from 80° flexion to 71° extension). When instructed to move between targets 30° apart, his mean amplitude was 46°; for 45° it was a 44°, and for targets 60° apart it was 80°.

### Conclusions

In conditions in which only minimal visual cues were available to allow him to judge the amplitude of the required movements, IW was able to only approximately scale the extent of his angular wrist movements, but could make repeated movements of a consistent amplitude. Under these circumstances he tended to reach a fixed extension limit of about 40°, with low variability, and was more variable in flexion.

## Experiment 2

### Aims

To test the deafferented subject’s ability to control movement amplitude and position without knowledge of starting position.

### Methods

#### Subject

IW was tested in this task in a single session which took place about 1 h after the completion of Experiment 1.

#### Task

The same equipment was used as previously described. In three blocks of trials, a single LED light was illuminated as a target located at one of two different fixed position on the arc, ±25° from the centre. On each trial, the subject’s wrist was slowly and passively moved to a random starting position; the experimenter shifted the subject’s wrist by moving the base of the handle, so that there was no direct contact with the subject’s hand or arm. IW was then instructed to move on a verbal cue to the target position, and to verbally signal that he had reached the target. The experimenter released the manipulandum just prior to the cue to move. This was repeated 10–15 times for each target position using a range of starting positions both left and right of the target. The subject was unable to see his wrist or hand throughout this experiment, and verbally confirmed that he had no knowledge of the starting position. No knowledge of results was given. As he had no visual feedback of his wrist, this was a test of his ability to use visual information about the desired end-point to control wrist movements without knowledge of the starting position.

### Results

**Fig. 3:**
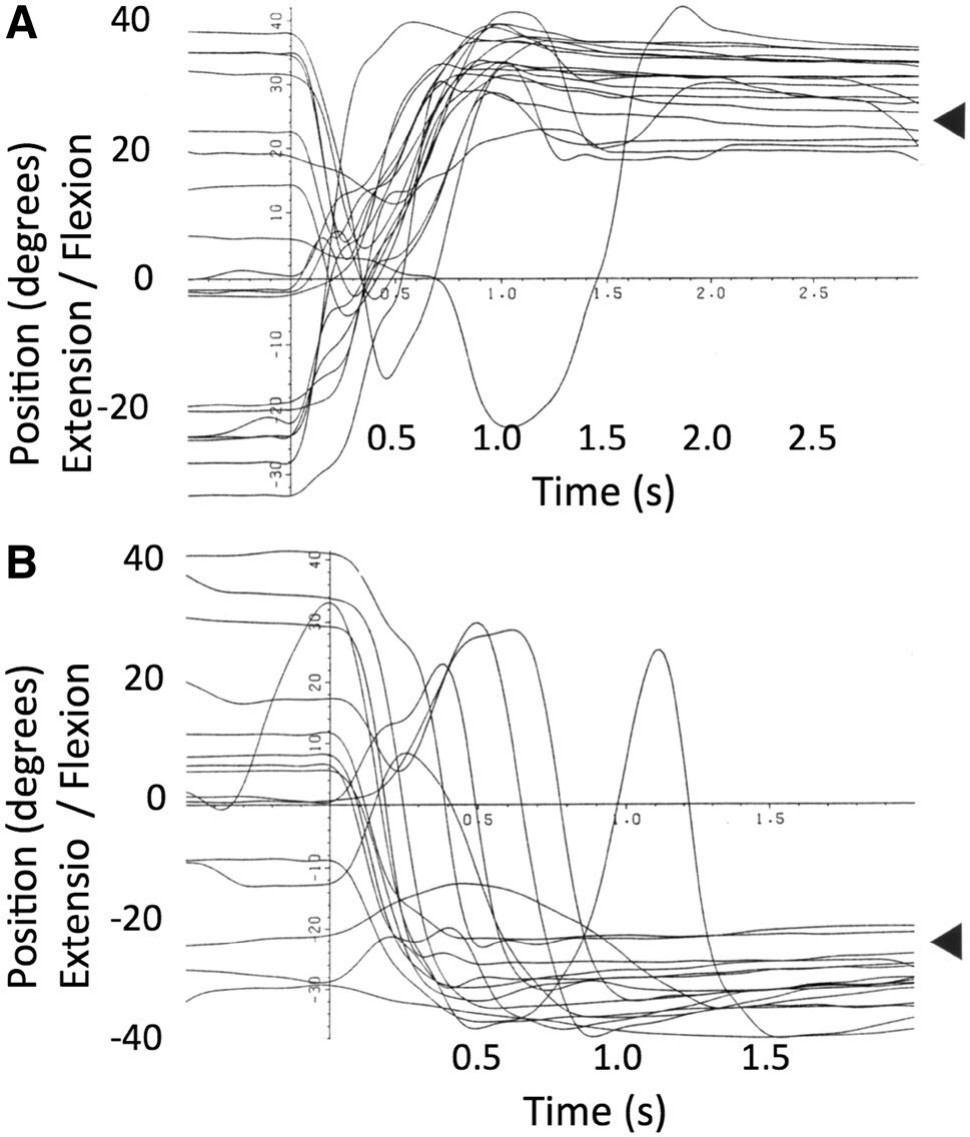
Experiment 2: records of wrist position during movements from unknown starting positions to fixed target positions 25° in flexion (**a**) and extension (**b**), indicated by the *arrow heads*. The subject was instructed to move to the target and verbally signal the end of his movement. All movements are shown, superimposed after alignment at the start of each movement. It is clear that the initial high variance of the wrist angle was greatly reduced by his movements, indicating successful approach towards each target position from a wide range of unknown starting positions

Figure 3 illustrates all movements recorded in this experiment, aligned by their start times. The arrowheads on the right indicate the target positions. IW clearly reduced the variance of his wrist angle during the movements, from their initial widely distributed start positions to positions clustered around the target position (mean absolute error 5.14°, SD of error 4.96°). He was not informed of his success, but on later questioning reported that he felt he had been able to achieve this task, although he was unable to explain how he had done so. However, examination of the data from this session suggested a possible strategy that the subject may have adopted. Figure 3a indicates that on many trials, the wrist angle did not move directly to the final target, but dipped towards a central position before a second, accurate, flexion movement was made to the target. The movements towards the extension target (Fig. 3b) were less consistent, some going directly to the final position while others showed first a flexion and then extension.

### Conclusions

This experiment suggests that despite absence of proprioceptive and visual feedback, IW could reach static target positions from unknown starting positions. The absolute error and variability (standard deviation) of the final errors are close to those reported by Marini et al. (2016) for participants with normal proprioception, in a very similar wrist flexion/extension repositioning task. They report a mean error of 4.8° compared to a mean of 5.14° for IW, and a variable error (SD) of 5.0°, very close to the 4.96° SD for IW. We suggest that IW may have been able to use a pre-learned strategy to reach a central position, and then move towards the final desired location. Experiment 1 had shown that he could voluntarily scale his movements. Hence knowledge that he had reached a fixed intermediate position might have allowed him a strategy to reach the final targets. Hypothetically, co-contraction of wrist flexors and extensors could bring the wrist central, and having commanded this movement, he could then make a scaled flexion or extension movement to the desired target position (Fig. 3a).

## Experiment 3

### Aims

To test the hypothesis developed from Experiment 2 that the deafferented subject was dependent on a strategy of moving first to a central wrist angle and subsequently to the desired target angle.

### Methods

#### Subject

IW was tested in this task in one session on the day following Experiments 1 and 2.

#### Task

This experiment differed from Experiment 2 only in the instructions given to the subject. He was instructed to ‘make a single movement directly to the target position’, and to again verbally signal that he had got to the target. The subject was unable to see his wrist or hand throughout this experiment, and verbally confirmed that he had no knowledge of the starting position.

### Results

**Fig. 4:**
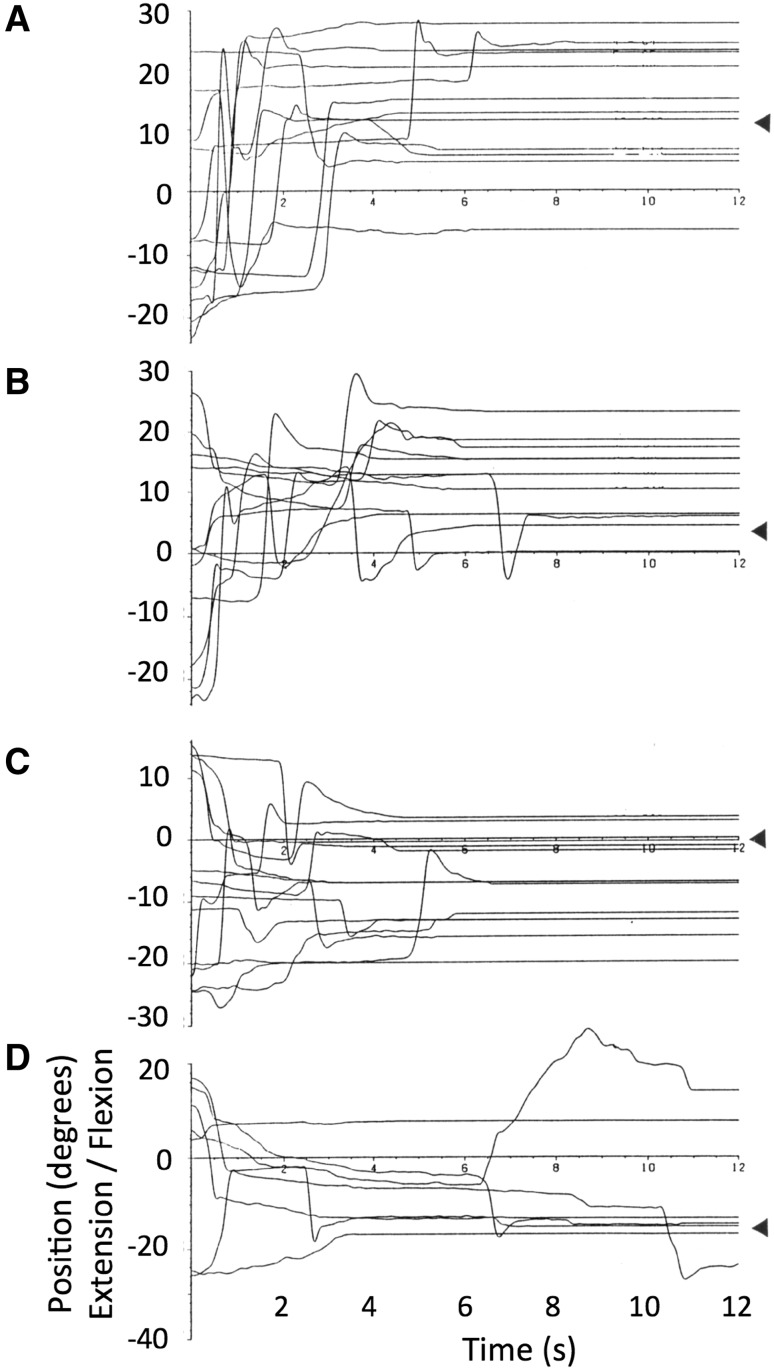
Experiment 3: records of wrist position during movements from unknown starting positions to four fixed target positions (indicated by the *arrow heads*). IW was instructed (but failed) to make a single movement directly to the target and to verbally signal the end of his movement. All movements are shown, superimposed after alignment at the start of each movement

Figure 4 illustrates all movements recorded in this experiment, again aligned by the start times. The arrowheads indicate the target positions, at approximately 12° and 5° in flexion (Fig. 4a, b) and 2° and 15° in extension (Fig. 4c, d). IW often clearly failed to make a single movement directly to the target, despite the instruction to do so. However, he also failed to reduce the final variance of his wrist angle compared to that achieved in Experiment 2. The mean absolute error across all four targets was 8.58°, 66% greater than in Experiment 2, while the standard deviation of the final errors was 13.87°, nearly three times more variable than in the previous experiment. Thus, the added task constraint, designed to avoid a strategy based on reaching an intermediate position, was sufficient to make this task impossible for the subject.

### Conclusions

We suggest that in the absence of visual or proprioceptive feedback, IW was unable to move his wrist directly to known target locations from an unknown starting position. Thus, in these conditions, he could not use a final position control strategy successfully for this single joint action.

Experiment 1 showed that while he could scale repeated movements with some accuracy, and repeat a sequence of several movements with maintained accuracy, this scaling was not precise, and the reproducibility of the final angles was much higher in extension than in flexion. However, Experiment 2 showed that he could position his wrist to specific targets with considerable accuracy, and in fact his accuracy is equal to intact participants (Marini et al. 2016). IW appears to adopt a strategy that might then be referred to as ‘via-point’ control, in that he moves from an unknown starting position towards at least one central position. When denied this strategy (Experiment 3) his variability increased threefold, and was considerably higher than in intact participants. Although we did not test the hypothesis in this experiment, it seems possible that there would be at least three such fixed via-points or ‘canonical positions’: the central one that we believe he used in Experiment 2, a second canonical position with the wrist flexed, and a third with the wrist extended, that might explain his extensor performance in Experiment 1. These three canonical positions could be reached by a simple motor control strategy in which fixed activation of wrist flexors alone, wrist extensors alone, or co-contraction of both muscles would be used. In support of this suggestion, it is clear from casual observation that IW often puts great muscular effort into movement, and he does hold his limbs in fixed postures with high stiffness when accuracy is needed (Cole 1995).

## Experiment 4

### Aims

To test the hypothesis developed from Experiment 3 that the deafferented subject IW may be able to reach one or more canonical positions of the wrist from an unknown starting angle using a strategy of final position control, whereas other positions of the wrist are reached using a strategy of amplitude control.

We argue that if these canonical positions are used, then movements towards them should be relatively accurate, despite uncertainty about the initial starting position. In contrast, movements to other positions, which we suppose are controlled by an amplitude control strategy, should reflect the intended movement amplitude. Nougier et al. (1996) developed the same hypothesis. We, therefore, aimed to compare amplitude and position control hypotheses using linear regression, predicting the position at the end of each movement from the error between the actual and instructed wrist position at the start. Amplitude control predicts a strong linear relation between the starting and final errors, with a unity slope, because the instructed amplitude should be added (with some noise) to the initial starting position, thus conserving the original starting error. In contrast, position control predicts a slope of zero, since variance in the start position should not affect the ability to reach the target position. However, to fully separate these differing predictions, it is necessary that the subject has some uncertainty about his wrist angle at the start of each movement. We, therefore, used a task involving long series of movements to defined targets made without visual feedback, in which we expected errors in actual wrist position to accumulate (Miall et al. 1995a; Nougier et al. 1996). In the start of any one movement during this sequence, the subject might assume that his wrist would be positioned on or near to the particular visual target aimed for during the previous movement. However, the actual wrist angle would be more variable and a measurable starting error would exist.

### Methods

#### Subjects

IW was tested in this task on two consecutive days. These tests took place several months after completion of Experiments 1–3. Seven control subjects were also studied on two separate occasions (age range 22–40 years, mean 27.5, SD 6.3; 5 male). These subjects had no known neurological deficits, and all were naïve to the purpose of the experiments.

#### Task

The same experimental set-up was used as described above. However, in this experiment, visual targets were displayed on a paper sheet placed on top of the board, thus blocking all vision of the hand and manipulandum, and the small fluorescent lamps were not used (Fig. 1b). A circular array of nine numbered targets was printed on a paper sheet, centred on the axis of the manipulandum, and positioned so that the targets were closely aligned with the arc of the moving manipulandum handle, with parallax errors calculated from the subject’s viewpoint of less than approximately 3°. Each target was separated by 15°, with a total range of 120°, which was slightly greater than the 95° range of his preferred comfortable movements in Experiment 1, but not at the anatomical limits of wrist movement (150°, see Experiment 5). The targets were numbered clockwise from extension to flexion (1–9, respectively) with target #5 corresponding to the position of the manipulandum reached when the wrist was aligned with the forearm. With the target sheet in place, the subject had no vision of hand, forearm or manipulator movement.

This experiment was conducted over six sessions, each lasting about 8 min, and including a sequence of 56 movements. For subject IW, three sessions were held on one day, separated by at least 30 min, and the next three held the following day, again separated by 30 min. In the intervals between these sessions the subject rested, took refreshments, or performed another unrelated task involving pointing to targets with a computer digitising pen. The control subjects completed all six sessions on 1 day, each separated by between 2 and 5 min.

Each subject was allowed to grip the manipulandum, and move it to the central starting position (target 5) with vision of the hand provided through the perspex window. Subjects were asked not to move the hand more than necessary to reach the target. The paper sheet displaying the targets was then placed over the window, obscuring all further visual feedback of the hand. The subjects then made a series of 55 wrist flexion or extension movements between nine visual targets (at 15° intervals between ±60°), without any vision of the hand or arm. Target numbers were presented verbally in a pseudorandom order at a rate of approximately one every 3 s. The order of targets was different in each of the six sessions, but was arranged so that the starting target was always target five, and that each of the nine target positions was reached from six different starting angles. In each sequence of 55 movements, the range of instructed movement was 45°–105°.

### Experiment 4a

A second test was held three months later used a slightly modified version of the experiment in which 82 movements were made in each of six sequences so that all nine targets were reached from all eight other possible targets. Only the control subjects completed this secondary experiment. This allowed movements between neighbouring targets 15° apart, as well as the maximum movement amplitude of 120°, whereas neither of these were included in the original experiment.

#### Analysis (Experiment 4 and 4a)

**Fig. 5:**
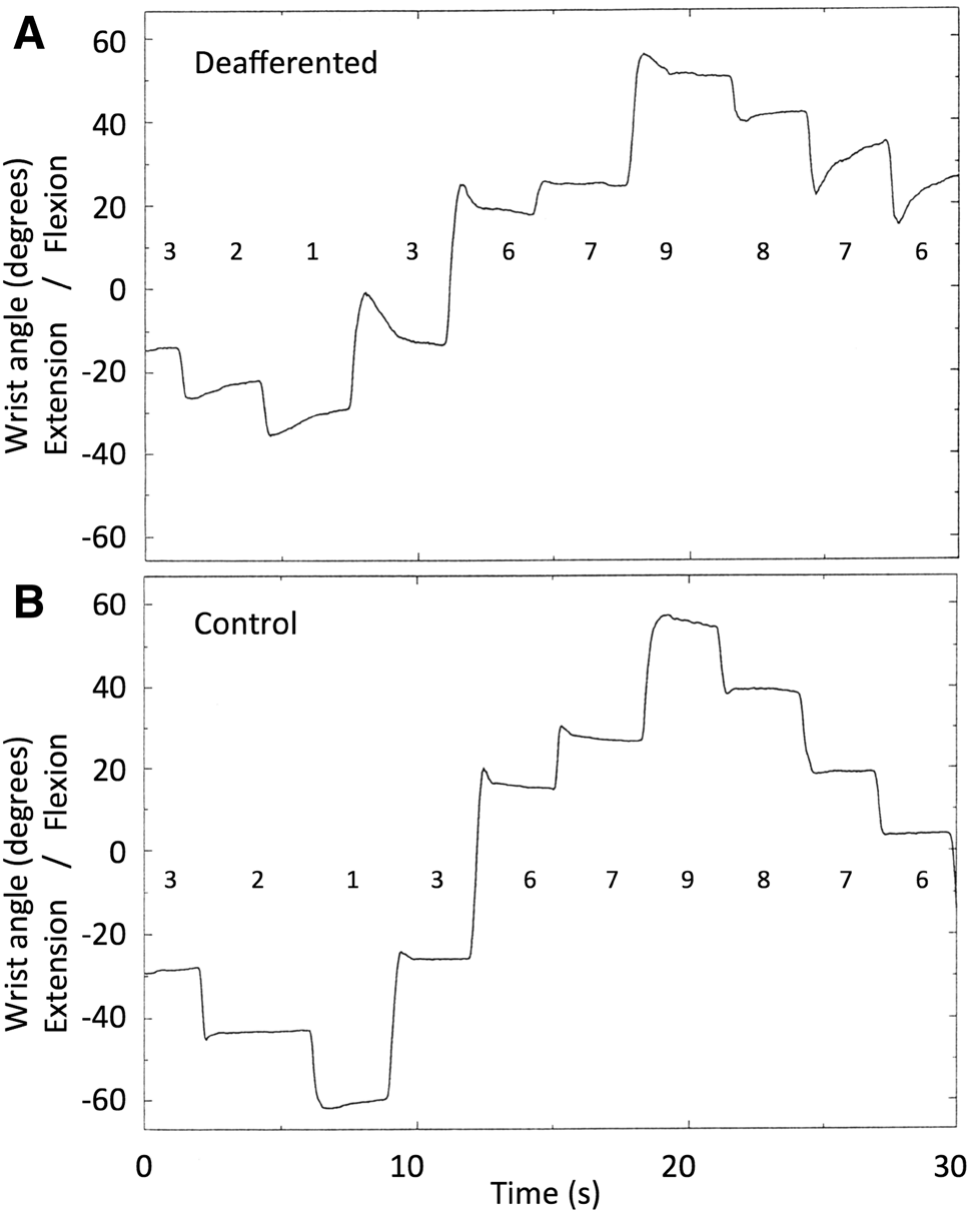
Typical movements made without visual feedback to a series of ten visually defined targets, by subject IW (*top*) and a control subject (*below*); these two series were collected as a part of Experiment 5, but are typical of the data in Experiments 4, 4a (controls only) and 5. The *horizontal row of numbers* indicates instructed targets (1 = 60° extension, 9 = 60° flexion)

The records of wrist angle (Fig. 5) were analysed using an interactive computer programme that first detected the large velocity peak corresponding to each movement, and then tracked backwards and forwards along the movement profile to find the points at which movement velocity dropped below 1°/s. These times were taken as the start and end of each movement, and the corresponding wrist positions recorded. All automatic movement selections were checked visually by the operator, and could also be compared to a timing marker recorded at the moment the subject verbally reported the end of each movement. Starting and final errors were calculated for each movement, and combined across the six sequences, giving six replicates of movement to each of the nine targets from six different starting targets. Linear regression equations were fitted across these six replicates for every combination of starting and final target positions, and the regression slope and goodness of fit (r-squared) noted. For the data collected from control subjects in Experiment 4a, movement amplitudes were also calculated with respect to the target amplitude and final errors calculated with respect to starting target positions.

### Results

#### Deafferented subject

**Fig. 6:**
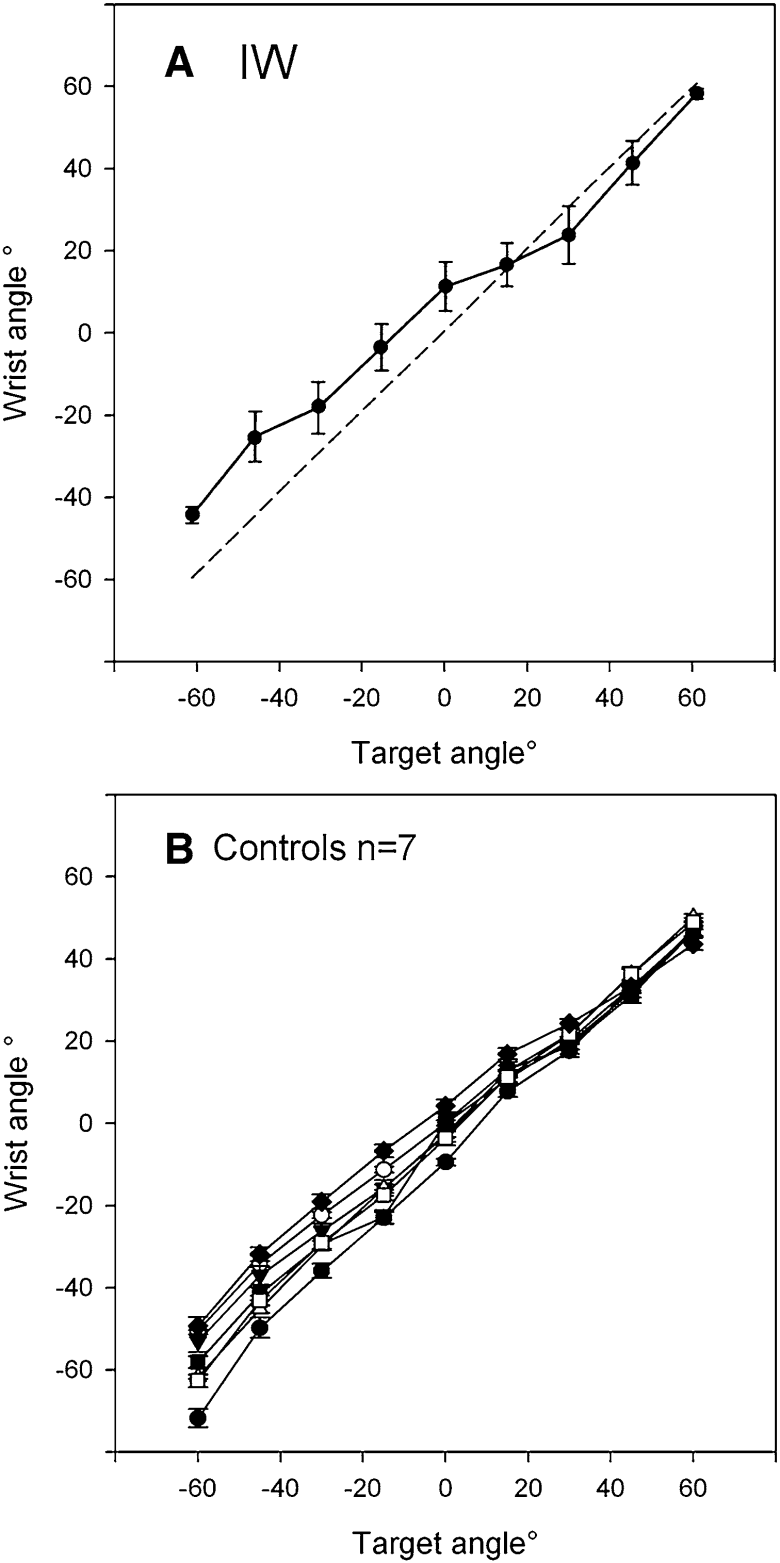
Experiment 4: final position accuracy of movements to the nine targets, for subject IW (*top*) and for seven control subjects (*bottom*). Note that the range of movement tended to be smaller than the target range, but that the relationship was approximately linear across all nine targets. In contrast, note also that the variance of IW’s final positions differed across the nine targets, being noticeably smaller for the extreme extension and flexion targets, 1 and 9, respectively. Target 1 is at −60° (extension) and target 9 is at +60° (flexion)

IW achieved reasonable positional accuracy, though the range of his mean final wrist angles was less than the ±60° range of targets (Fig. 6a). The variability of final wrist angles was noticeably lower for the targets located at extreme flexion and extension (targets 1 and 9) than for intermediate targets (targets 2–8).

**Fig. 7:**
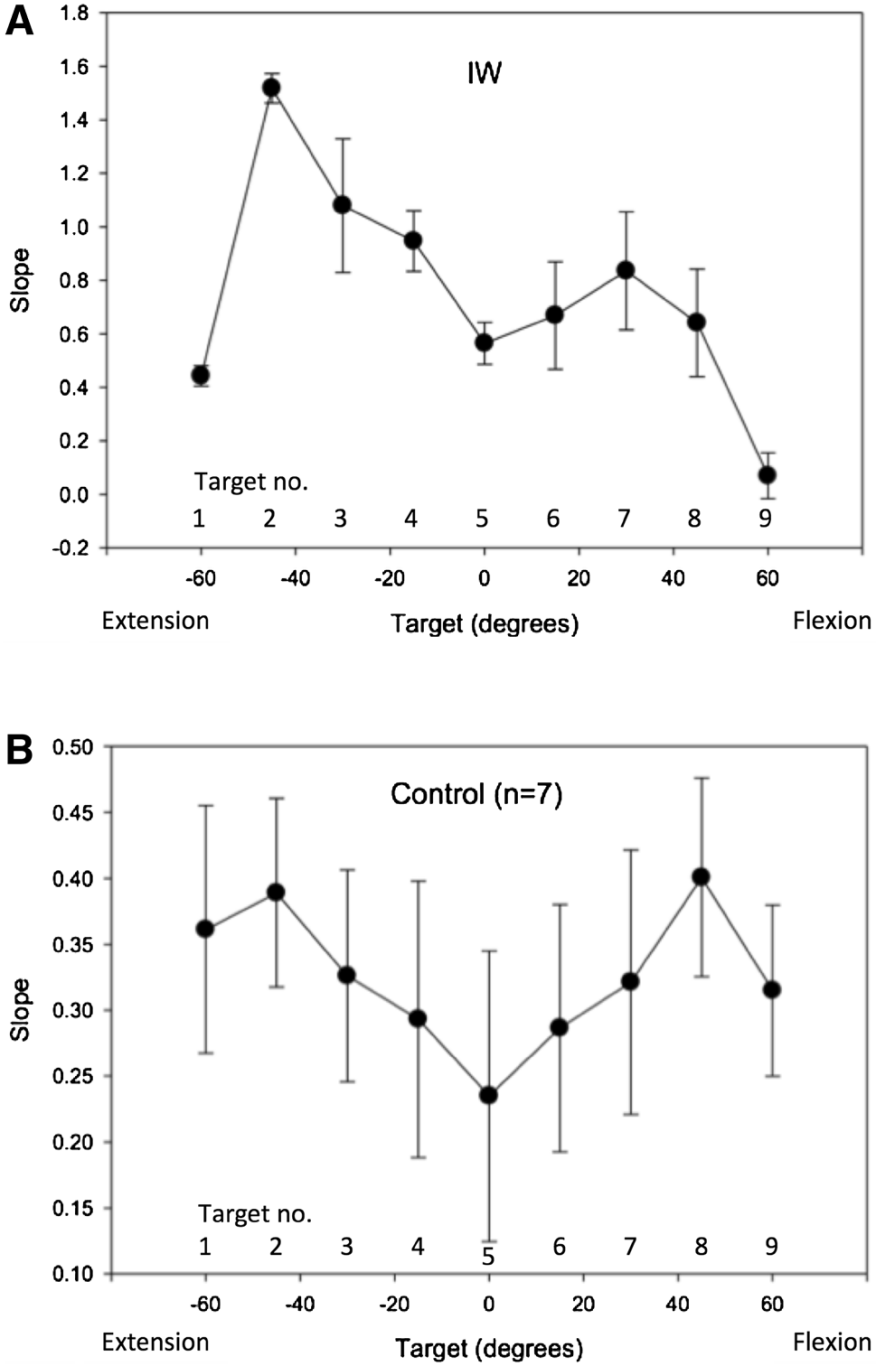
Experiment 4: the mean slope of regression lines between starting and final errors during movements towards each of nine targets. Each regression line was fitted to six replicates. **a** The data from Subject IW; **b** corresponding group mean data from seven control subjects. *Error bars* represent SD. Target 1 is at −60° (extension) target 5 and target 9 is at +60° (flexion)

For movements towards targets 2–4 and 6–8, the regression slope between starting error and final error was close to or above unity (mean = 0.948; Fig. 7a), suggesting an amplitude control strategy. However, movements to the extreme extension and flexion targets, 1 and 9, showed a lower slope (mean = 0.26) suggesting position control. Movements to target 5, which places the hand approximately central in front of the forearm, also tended to have a lower slope than other intermediate targets. A one-way ANOVA showed these mean slopes were statistically different [*F*(8,46) = 6.84, *p* < 0.0001]; post hoc comparisons showed the slope for the extension target 1 was smaller than for target 2; the central target 5 was smaller than for target 2; and the flexion target 9 was smaller than for target 2, 3, 4 and 6. In addition, the slope for movements to target 2 was higher than for targets 6 and 8 (all *p* < 0.03, Tukey’s HSD corrected). This implies that for this target, errors were amplified during the action, so that end errors were about 50% larger than starting errors.

#### Control subjects

The seven control subjects also showed reasonable positional accuracy, although the mean errors were still significant (Fig. 6b). The mean regression slopes showed a similar pattern to that shown by IW, with lower slopes at the extension and flexion extremes, and at the central position (Fig. 7b), but the differences were not statistically significant [*F*(8,48) = 0.414, *p* = 0.91]. Four of the seven controls showed a clear minimum at the central target, and a fifth control showed a minimum at −15°. Individuals often showed a low regression slope for movement towards one extreme target or the other, but this was not evident in the group average.

A mixed ANOVA contrasting the within participant factor of regression slope across the nine targets against the between participant factor of group (the seven controls against IW) demonstrated a main effect of target [*F*(8,48) = 3.49, *p* = 0.03], a main effect of group [control vs. IW: *F*(1,6) = 66.8, *p* < 0.0001], and a significant interaction [*F*(8,48) = 3.02, *p* = 0.008]. Post hoc *t* tests between the controls and IW demonstrated that the interaction was driven by significant differences at targets 2 and 3 (*p* = 0.001 and *p* = 0.016, respectively; all others *p* ≥ 0.070).

### Experiment 4a

**Fig. 8:**
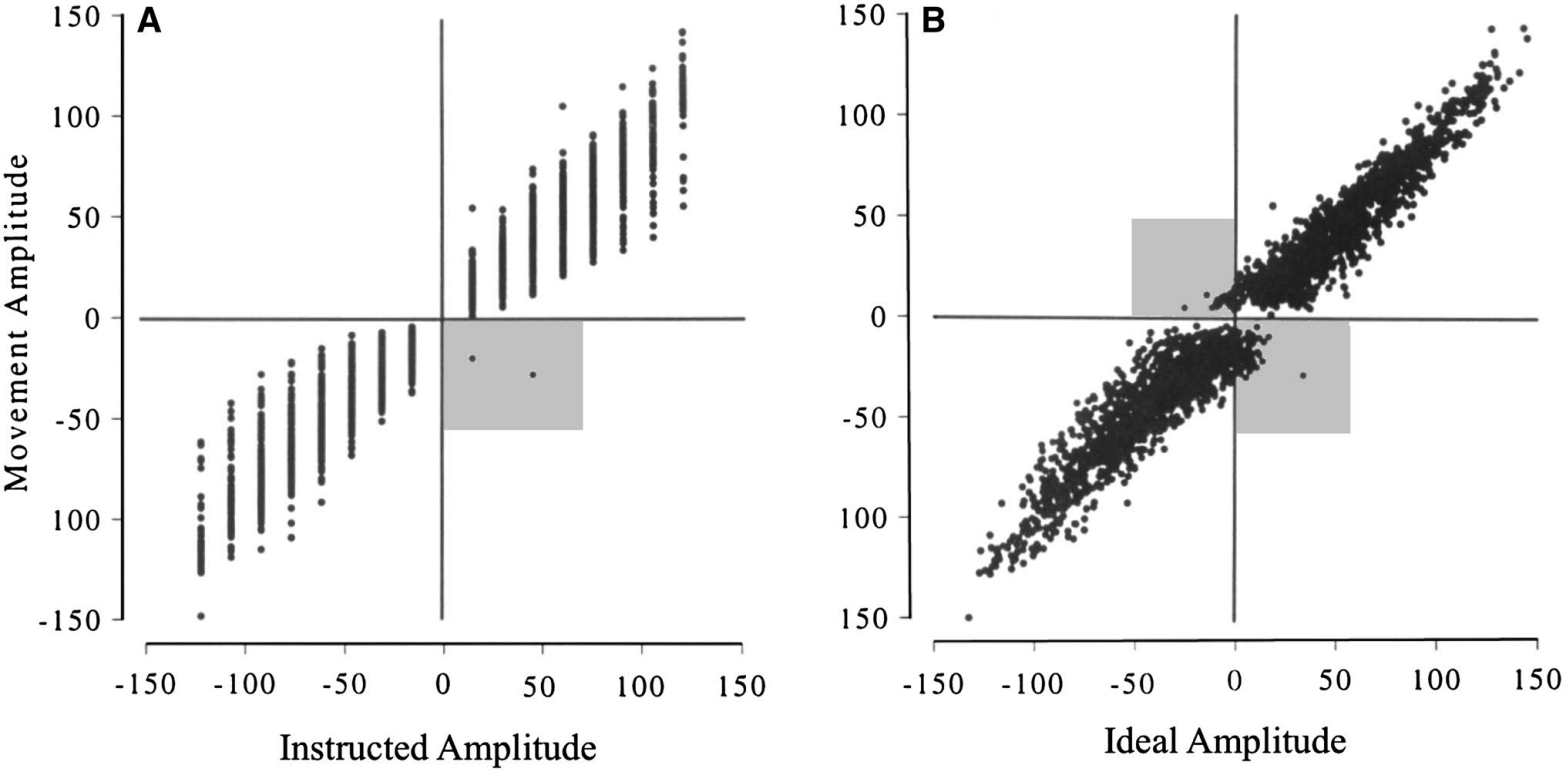
Experiment 4a: *scatter plots* of movement amplitude against the instructed or ideal amplitude for control subjects. **a** For each movement, the instructed amplitude was taken as the angular distance between the previous target angle and the subsequent target. Note that for the control subjects there were only 2 out of 3086 movements whose direction conflicted with the instructed direction (*grey area*). **b** The same movement amplitude data (*vertical axis*) plotted against the ideal amplitude, calculated as the distance between the actual starting position and the instructed target position. Movements of the ideal amplitude would result in zero terminal errors. In total, 85 movements (2.75% as highlighted by the *grey areas*) were made in the wrong direction to reduce the error

In a repetition of this experiment, tested for the seven control subjects only, the original pattern was replicated, but again without clear statistical differences in regression slope across the nine targets. Importantly, however, this new series of movements included steps of only 15°, and this allowed us to distinguish movements made in the instructed direction from those in the direction to reduce positional error. For example, an instruction to move from target 4–3 requires wrist extension. However, if the wrist was already beyond target 3, due to errors in the previous movements, then the wrist should actually be flexed to accurately reach the correct position. The control subjects always moved in the instructed direction (with only two exceptions out of 3086 trials, Fig. 8a). In contrast, 85 of these small movements actually increased the positional error (Fig. 8b, grey zones). In total, on 2.75% of all trials, which represent 11% of the 15° amplitude trials, control subjects moved in the wrong direction to reduce their positional error.

### Conclusions

We conclude that in the absence of visual feedback, the deafferented subject IW often used a movement strategy consistent with amplitude control to move the hand, but that for canonical positions in extension and flexion, and in the central wrist position, a strategy more consistent with position control may have been used. Terminal variability was reduced at these canonical positions, but mean accuracy was still poor, which suggests that IW’s ability to effectively balance efferent commands was imperfect. This is highlighted by movements towards target 2, where the slope of the regression between start and end errors was positive, implying he amplified movement errors in this condition, which consistent with (inaccurate) amplitude control, and inconsistent with position control.

For the control subjects, a tendency towards the same result was seen, in that the mean regression slopes were somewhat lower at the central target positions than at other positions (Fig. 7b), but this trend did not reach statistical significance. However, the mean slope for the controls was significantly lower compared to IW (overall control mean 0.325 vs. IW’s overall mean of 0.75). Thus, the control subjects appear more reliant on a position control strategy than on amplitude control, and appear to use position control especially near the central target. In some respects this result is unsurprising, as the controls had intact proprioceptive information about hand position, and thus would be expected to display behaviour consistent with a position control strategy; that is, they should indeed take account of the starting errors in calculating the amplitude of the next movement (van den Dobbelsteen et al. 2001).

Finally, we note that the terminal errors for the control subjects were quite significant (Fig. 6b). In fact, the mean size of their errors was not much different from the deafferented subject, although the variance of their final positions was smaller (Fig. 6). This is important because, had they been very accurate, it would have invalidated our analysis method: had there been negligible errors at the start and end of each movement, then the regression-based analysis could not differentiate between amplitude and position control.

However, in the repetition of this experiment for control subjects (Experiment 4a), there was good evidence that their smallest movements were made independent of starting error: if the subject was instructed to move clockwise from the one target to the next, the wrist movement was always clockwise, even if an anticlockwise movement was necessary to reach the target (Fig. 8b). Thus, they moved to obey the implied target direction, and conserved or even increased positional errors, rather than moving to violate the directional instruction and reduce their error. Note that for larger movements, and for all movements in the first part of Experiment 4 (for IW and the control subjects), the starting errors were always smaller than the smallest instructed movement amplitude of 45°.

We conclude that for the control subjects, their behaviour was consistent with position control, albeit with a cognitive supervision of this task that imposed directional (and thus amplitude) control for the smallest movements. It might be expected that the same argument would apply to subject IW, as it is clear that he is very heavily reliant on the visual aspects of any task that he completes; hence, we might expect that his movement vector would closely reflect the instructed vector between start and target positions. Sarlegna et al. (2006) have shown the importance of visual target vectors on corrective movement for the deafferented subject GL, in whole arm movements towards targets that jump laterally. However, from our regression analysis, we suggest that while IW uses a vectorial amplitude control strategy for most movements, he has adopted a end-point position control strategy to reach three ‘canonical’ wrist positions. These three positions are consistent with a control strategy of reproducible levels of flexion and extension, and of co-contraction of his wrist muscles for the middle point.

## Experiment 5

### Aims

To test the hypothesis that IW used a simple ‘on–off’ control strategy of muscle contraction to reach the three canonical positions, by recording wrist flexor and extensor surface EMGs during wrist movement.

### Methods

#### Subject

IW was tested in this task on two consecutive sessions, separated by 1 h. The occasion was approximately 3 years after completion of Experiments 1 and 2.

#### Task

The same experimental set-up, behavioural task and protocol was used as described above (Experiment 4). Surface EMGs were recorded from superficial wrist flexor and extensor muscles over the subject’s left forearm (Fig. 1b). EMG signals were recorded with isolated DC pre-amplifiers (CED 1902), digitally low-pass filtered and sampled at 1 kHz. The manipulandum position was also sampled and stored at 1 kHz. Digitised data were then analysed using Matlab, first rectifying the data, and then using a fourth order, zero phase Butterworth low-pass filter of 20 Hz. The rectified and filtered EMG was averaged across all movements towards each final target position, and the area under the average curves measured for 100 ms before to 400 ms after the onset of movement.

At the end of the sequence of trials, IW was instructed to move from a relaxed position near the centre to the extremes of flexion and extension, beyond targets 1 and 9, and repeated each movement twice.

### Results

**Fig. 9:**
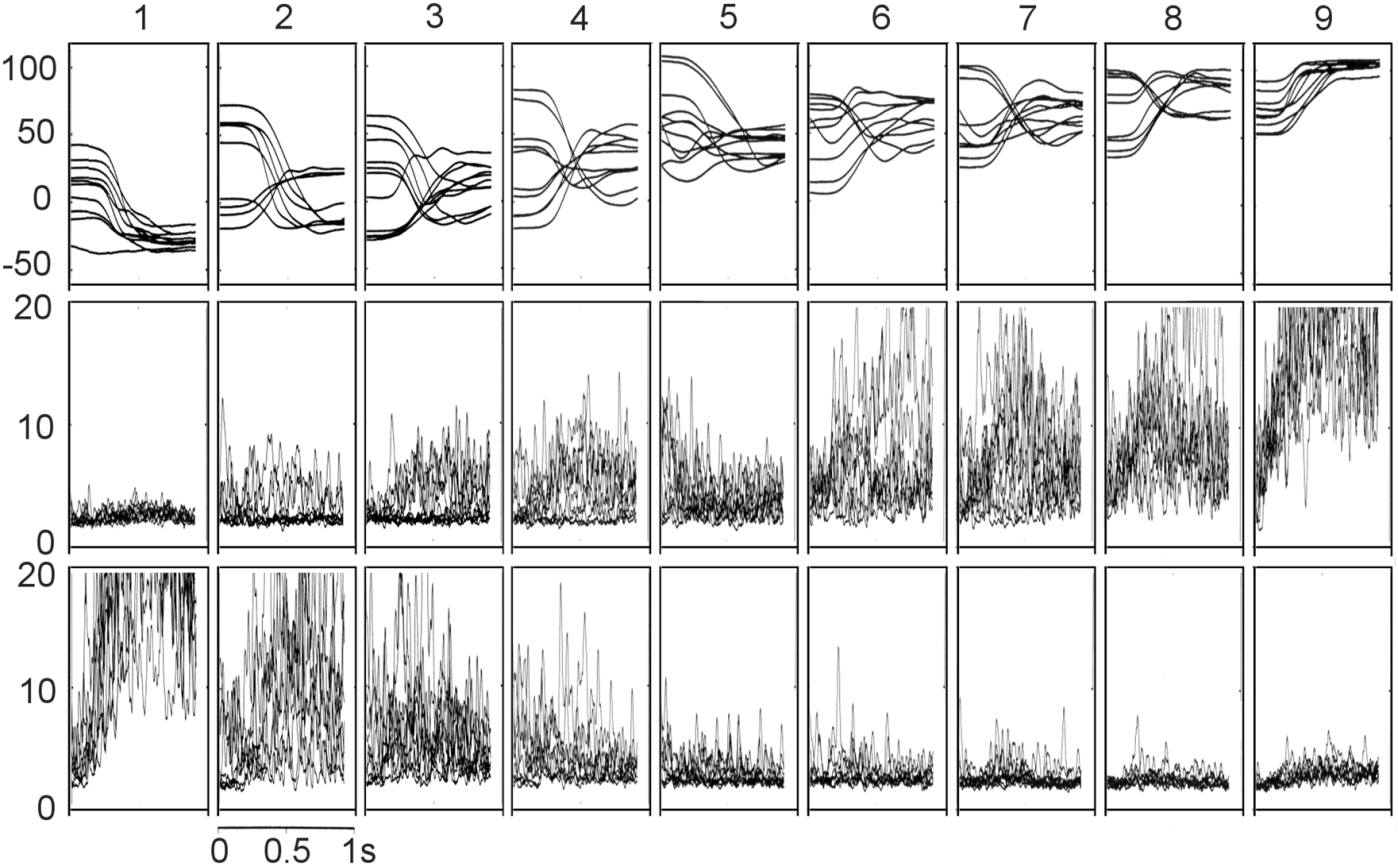
Typical EMG records from IW’s superficial flexor and extensor muscle groups during movement to each of the nine targets, from various start positions. *Top row* movement traces (wrist angle in degrees against time) for each trial. Target 1 is 60° in extension, target 5 is central and target 9 is 60° in flexion. *Middle* wrist flexor EMG records from flexors (uV against time) superimposed for ten trials per target. *Bottom* superimposed EMGs records from wrist extensor muscles

**Fig. 10:**
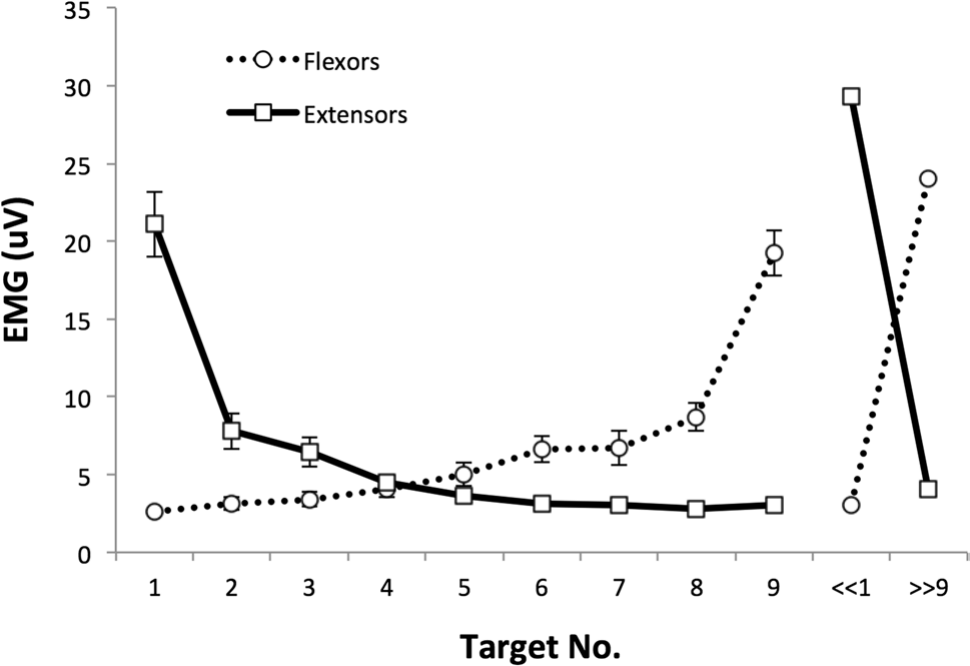
The average flexor and extensor muscle activity in movements to each of the nine target positions, from extension (target 1) to flexion (target 9). Each point is the mean area under the rectified and smoothed surface EMG signal for a period 100 ms before to 400 ms after the start of wrist motion (see Fig. 9). *Error bars* are the SEM (*n* = 11–15). At the right is the mean extensor and flexor activity when IW reached to his extremes of wrist extension and flexion in the apparatus (*n* = 2 trials each)

Figure 9 shows typical EMG records from IW’s wrist flexor and extensors during movement towards all nine visual targets, and Fig. 10 shows the mean areas under flexor and extensor average EMG records. The mean wrist angles achieved in reaching to each of these nine targets were similar to those reported in Experiment 4 (Fig. 6a), with an undershoot of targets 1 and 2 (in extension) and slight overshoot of target 9, in flexion. These EMG results suggest that IW reached targets 1 and 9 by strong activation of wrist extensor or flexor muscles, respectively, and with negligible activation of the antagonist (Fig. 9 left and right columns). The mean extensor EMG (Fig. 10) was significantly higher for target 1 than for all other targets (*p* < 0.0001, corrected for multiple comparisons with Tukey’s HSD); the mean flexor EMG was significantly higher for target 9 than all others (*p* < 0.0001). At all intermediate target positions, there was activation of both flexors and extensors, to differing degrees. Hence the relative proportion of flexor and extensor activity to reach all other targets (2–8) changed in a graded fashion, with less activation of either flexor or extensor muscle groups when moving towards target 5.

When asked to reach his flexion and extension limits in the same apparatus, IW demonstrated that he could flex his wrist to 80° and extend it to 71° (151° total, compared to the ±60° target range). The recorded EMG activation in flexor and extensor muscles was maximal in these conditions (Fig. 10, right side). Because of the limited EMG data (two trials only per condition), statistical comparisons with trials reaching to the visual targets were not attempted.

Finally, we tested when the antagonist muscle activity became significant. In trials to extension targets, there was no significant difference between the flexor activity seen in reaching target 1 compared to targets 2–5 (*p* < 0.05, Tukey’s HSD). Flexor activity became significant for target 6 (*p* = 0.049, Tukey’s HSD) and beyond. The reverse pattern was seen for extensors: activity was not significantly different from target 9 compared to targets 4–8 (*p* > 0.5, Tukey’s HSD), and only became statistically significantly elevated when reaching towards targets 2 (*p* = 0.004) and 1 (*p* < 0.0001). There was a trend towards significance for target 3 (*p* = 0.114).

### Conclusions

The hypothesis that the three canonical positions were reached by strong activation of flexor and extensors was partially supported by this experiment. We saw powerful contraction of wrist extensor or flexor muscles to reach targets 1 and 9, respectively, as predicted, significantly higher than when reaching to any of the other targets. However, our prediction that the central target 5 would be reached with co-contraction was not supported, as there was very little activation of either flexor or extensor muscles in these movements, not significantly elevated from the minimal levels seen in the antagonist when reaching to the lateral targets 1 and 9, respectively. Thus, a modified version of the original hypothesis seems to be justified: that strong contraction of one or other muscle group is used to reach the two extreme canonical positions. In principle, then, a strategy of co-relaxation could be used to reach the central position, since during complete relaxation the wrist lies in this mid position. There is some evidence of slight relaxation in flexion in trials towards target 5 (Fig. 9, middle row, middle panel). However, there were small and sustained EMG signals in movements to this target (Fig. 10), albeit not statistically elevated. We will return to this in the general discussion.

## General discussion

We present data from a series of experiments with IW, a well studied man who has spent decades exploring how to recover control of his movements after suffering a near complete loss of sensation from below the neck at the age of 19. Without proprioception, and in circumstances in which he was able to repeatedly make wrist flexion and extension movements without any visual feedback, we conclude that he has adopted a strategy of ‘canonical control’, in which three canonical wrist positions can be reached from unknown starting locations with a strategy based on fixed activation of flexor or extensor muscles. The mid position was reached by minimal contraction of both flexors and extensors, at a level seen in extensors during extreme flexion and in flexors during extreme extension, (neither muscle was ever completely silent during this task). Thus, rather than co-contraction of flexors and extensors he appears to reach the mid position by relative relaxation. However, in this modified version, the advantages of the original scheme remain, in that IW needs use only a rather simple control of muscle forces to reach these three canonical positions.

Interestingly, the graded ratio of flexor and extensor activation seen in the averages of movement towards other target positions implies reasonable control of the contraction of each muscle group. However, there was considerable scatter between recordings made on individual trials when reaching to the same target from similar starting positions. Coupled with our earlier evidence of quite consistent movements to and fro between two wrist angles (Fig. 2), and yet of large errors in final wrist position when the start position is unknown (Fig. 4), these data imply that IW can control muscle forces, but in the absence of information about his starting position, this control is not sufficient to allow good positional control to reach all nine target wrist angles. Instead, he can reach the lateral positions (targets 1 and 9) with relatively high reliability (as indicated by the low variance in terminal errors at these positions, Fig. 6a), and can also reach to a known central position with a strategy biased towards position and not amplitude control (Fig. 7a). For all intermediate wrist positions, he appears to be uncertain of his starting position, and adopts an amplitude control strategy to move towards other targets.

We speculate that the position control mode used for the extreme and central targets may reflect a combination of two factors. The first is the mechanical characteristics of the muscle-joint system. The wrist joint has a limited range of movement. Thus, any flexor or extensor torque greater than a certain level will bring the joint to a fixed flexion or extension position, and as one approached the anatomical limit there is a reduced relation between EMG level and final position. These anatomical constraints mean that accurate positioning at extreme targets can be achieved even with relatively inaccurate motor control. Note that while targets 1 and 9 were not the extremes of IW’s wrist motion, the agonist EMG activity in reaching them was statistically higher than for all eight other targets. In principle, an anatomical account might also account for the low variability of central targets. In the absence of any voluntary muscle contraction, joints typically revert to a canonical position which depends on the passive elasticity of the surrounding tissues, gravity, and other action-independent factors. In principle, IW could identify that one of the central targets corresponded to this canonical wrist position, and then he could rely on passive mechanics to bring the joint to rest in this position. When subsequently asked about reaching position 5, IW said that he had tried to relax all muscles, since he knew that this would lead to the wrist being in neither flexion nor extension. He also reported that positions 1 and 9 were easier to reach because he visualised them more clearly, suggesting that his visual representation of them might have helped in selection of just flexor or extension activity. Figure 9 shows that EMG level remained elevated even when the wrist came to rest in the central target position, showing that IW did not, in fact, use a strategy of complete relaxation. Instead he might be able to match the low levels of motor commands to flexor and extensor muscles, which would also bring the joint to the canonical position. In fact the mean activation of flexor and extensor muscles was most closely matched at target 4, neighbouring target 5: but bearing in mind the challenge of calibrating muscle force with surface-recorded EMG, we cannot rule out that he was commanding low level matched signals to flexors and extensors to reach target 5. In summary we believe IW uses a strategy based on end-point positional control to reach three canonical wrist angles, and used amplitude control for other target angles.

Nougier et al. (1996) also argued for amplitude control in GL, another deafferented subject, but did not subdivide their analysis in a way that would have shown canonical positions. Nougier’s work is consistent with Bock and Eckmiller (1986), Ghez et al. (1995) and with the recent work of Marini et al. (2016) who argue for amplitude control in normal participants. In contrast, Walsh et al. (1979) argued that neurologically intact participants used a blended strategy in making reaching whole arm movements to targets, with both target distance and location influencing accuracy, while Hudson and Landy (2012) and Graaff et al. (2016) found evidence for both strategies. There is also evidence that the vector control strategy is prone to central neurological loss following stroke, leaving greater reliance on position control (Rashbaum et al. 2015). Serial strategies have also been proposed (Scheidt and Ghez 2007; Yadav and Sainburg 2011) in which movements are achieved by initial vectorial control but terminated under final positional or impedance control. At the other end of the spectrum, Bizzi and colleagues (Bizzi et al. 1976; Polit and Bizzi 1979; Latash and Gottlieb 1992, 2010) argued for equilibrium- or end-point positional control, which in the intact system can allow achievement of “equifinal” positions despite unknown start locations and/or perturbations during the movement. Feldman has highlighted that both surgical and pathological deafferentation remove spinal reflex circuits, such that simple equilibrium control would be limited in these circumstances (Feldman and Latash 2004).

It is, however, instructive to acknowledge that IW, after many years of active experimenting with his own movements has still only limited ability to control single joint wrist flexion and extension movements accurately. We have shown here that he appears to do so with a pragmatic strategy that makes use of the simple relationship between wrist angles and fixed, and for the two lateral positions, powerful flexor and extensor muscle activation to find intermediate “via-points” or canonical positions. If allowed to reach these, he can then make amplitude-controlled movements towards other desired angles. This implies some control of movement amplitude, by commanding muscle forces (Experiments 1–3) but with limited ability to finely regulate or internally monitor efferent signals [Experiments 4 and 5; see also Miall et al. (2000), Cole and Sedgwick (1992), and Miall and Cole (2007)]. While it is possible that IW may have some sense of extreme joint angles from remaining small fibre axons, it seems more likely to us that he has learnt that a high flexor or extensor force, even if not finely controlled, would bring his wrist to a consistent final angle. Decisions about loads on his wrist, that might be based on peripherally originating perceptions, are very inaccurate (Miall et al. 2000). Graaff et al. (2016) showed that neurologically intact participants are biased towards a positional strategy during repeated movements, but only if repeating reaches to fixed target position, allowing for learning. Improved accuracy of movements made from known starting locations has been reported before for deafferented participants (Ghez et al. 1995; Miall et al. 1995a) and suggests a short term visual recalibration of their motor control. Thus, the canonical control that IW appears to adopt for these single joint wrist movements is a pragmatic solution to reaching specific joint angles, despite the lack of spinal reflexes or accurate internal monitoring of commanded forces. We cannot say whether the strategy of reaching canonical positions with simple “on–off” muscle activation could be generalised to other, multi-joint actions. Examples might include flexion of the elbow and wrist to “locate” and recalibrate the arm against the chest, or adduction of the shoulder to bring the arm against the trunk. It may also form a component of the simplifying strategy provided by muscle synergies when attempting high dimensional, mutli-joint control (Todorov and Jordan 2002). The synergies are selected to isolate control noise task into irrelevant dimensions, and so less accurate control of force is required in those dimensions.
